# An Atypical Descending Variant of Guillain–Barré Syndrome With Bulbar Palsy, Autonomic Instability, and Delayed Colonic Pseudo-Obstruction: A Case Report

**DOI:** 10.7759/cureus.95802

**Published:** 2025-10-31

**Authors:** Tarun A Sahu

**Affiliations:** 1 Critical Care Medicine, Asian Institute of Gastroenterology Hospitals, Hyderabad, IND

**Keywords:** acute colonic pseudo-obstruction, atypical gbs, autonomic disturbance, bulbar palsy, descending paralysis, gbs, guillain- barré syndrome, ogilive syndrome

## Abstract

Guillain-Barré syndrome (GBS) is an acute, immune-mediated polyradiculoneuropathy that is the most common cause of acute flaccid paralysis worldwide. The classical form manifests as an ascending, symmetrical weakness, but atypical variants, including descending presentations, have been increasingly recognized. Such variants often pose diagnostic challenges and are associated with more severe disease and prolonged recovery. We describe a 70-year-old woman with well-controlled hypertension who presented with progressive dysphagia, pooling of saliva, and upper limb weakness. Clinical examination revealed bulbar dysfunction, areflexia in the upper limbs, and motor power in the upper limbs was markedly reduced to grade 1/5 on the Medical Research Council (MRC) scale, while lower limb strength was preserved. Within hours of admission, the weakness progressed to quadriplegia with respiratory distress, necessitating intubation and mechanical ventilation. Cerebrospinal fluid analysis demonstrated albumin cytological dissociation, and nerve conduction studies revealed changes consistent with GBS. Intravenous immunoglobulin was initiated but discontinued due to anaphylaxis; therapeutic plasma exchange was subsequently performed. The patient had marked cardiovascular lability and, after one month, acute colonic pseudo-obstruction (ACPO), which resolved with conservative management. Around the 40th day, she experienced complete left lung collapse due to mucus plugging, which was successfully managed with bedside bronchoscopy. Persistent bulbar dysfunction delayed decannulation until day 80. Remarkably, full neurological recovery was achieved only after six months of follow-up. This case highlights several atypical and severe features of GBS: descending onset with bulbar involvement at presentation, multisystem autonomic dysfunction extending to the gastrointestinal tract, and a protracted course despite early immunotherapy and supportive care. The unusually delayed onset of ACPO and persistent bulbar palsy underscores the need for vigilance beyond the acute phase. Hence, clinicians should maintain a high index of suspicion for atypical variants of GBS in patients with acute, progressive weakness, even when the presentation deviates from the classical ascending pattern. A structured, multimodal diagnostic approach and timely initiation of therapy are essential to prevent complications. Severe variants may follow a prolonged recovery trajectory, requiring sustained multidisciplinary management.

## Introduction

Guillain-Barré syndrome (GBS) is an acute, immune-mediated polyradiculoneuropathy that affects the peripheral nerves and nerve roots and represents the most common cause of acute flaccid paralysis worldwide [1]. It typically presents with rapidly progressive limb weakness, areflexia, and variable sensory involvement, most often following an infection such as *Campylobacter jejuni* gastroenteritis [2]. Although the classical phenotype is an ascending, symmetrical paralysis beginning in the lower limbs, a broad spectrum of atypical variants has also been recognized, including rare descending forms with prominent bulbar involvement [3,4]. This clinical heterogeneity, together with frequent overlap of motor, sensory, and autonomic features, can obscure early diagnosis and complicate management [1]. Among these, autonomic dysfunction is particularly important, occurring in up to two-thirds of patients and manifesting as cardiovascular or gastrointestinal instability [5]. Prompt recognition of such atypical patterns is essential, as they are often associated with severe disease requiring ventilatory support and prolonged rehabilitation [6].

We report a case of a 70-year-old woman who presented with a descending variant of GBS marked by early bulbar dysfunction, severe autonomic instability, and the unusual complication of acute colonic pseudo-obstruction (ACPO). Her clinical course highlights the diagnostic and therapeutic challenges of atypical GBS, while underscoring the importance of timely recognition and appropriate management in achieving favorable outcomes.

## Case presentation

A 70-year-old woman with a history of well-controlled hypertension was admitted to the emergency department with progressive dysphagia to both solids and liquids, along with pooling of saliva. She reported numbness and tingling sensations (paresthesia) and progressive weakness in both upper extremities over two days. On examination, she was afebrile, alert, and oriented. Her vital signs included a heart rate of 120 beats per minute, blood pressure of 230/120 mmHg, respiratory rate of 20 breaths per minute, and oxygen saturation of 97% on room air. Neurological examination showed impaired gag and cough reflexes and absent deep tendon reflexes in the upper limbs. Upper limb motor strength was reduced to grade 1/5 on the Medical Research Council (MRC) scale, while sensory function remained intact. Examination of the lower limbs was unremarkable, with normal motor power and reflexes. Cranial nerve evaluation revealed no facial weakness or ophthalmoplegia. No additional abnormalities were detected on general or systemic examination. Baseline laboratory investigations, including complete blood count, renal and liver function tests, thyroid profile, and serum vitamin B12 and folate levels, were within normal limits. On further inquiry, it was revealed that she had experienced acute diarrhea about two weeks earlier after eating at a local restaurant.

Within eight to ten hours of hospital admission, however, her condition deteriorated rapidly, with the weakness extending to the lower extremities (MRC grade 1/5) and accompanied by acute respiratory distress with desaturation. In view of impending respiratory failure, she was promptly intubated and initiated on mechanical ventilation. Magnetic resonance imaging (MRI) of the brain and spine was done, which revealed no significant abnormalities. Cerebrospinal fluid (CSF) analysis showed albumin cytological dissociation, with mildly elevated protein concentration and normal cell count, while all other parameters were within reference limits (Table 1).

**Table 1:** Examination of CSF

Macroscopic Examination		Reference Range
Aspect	Clear	Clear
Color	Transparent	Transparent
Chemical Examination		
Proteins	59 mg/dL	15-45 mg/dL
Glucose	90 mg/dL	45-95 mg/dL
Microscopic Examination		
Total cell count/µL	5	0-5 cells
Cell type	All lymphocytes	Mainly lymphocytes

In addition, electrophysiological evaluation with nerve conduction velocity (NCV) studies revealed diffuse motor conduction slowing in all four limbs, prolonged distal motor latencies, and absent F-wave responses. The original NCV tracings were unavailable for inclusion in this report; however, the findings were interpreted and documented by a certified neurologist. Testing for anti-ganglioside antibodies could not be performed due to the non-availability at the treating institution. In conjunction with the clinical presentation, these findings were consistent with the diagnosis of an atypical variant of GBS.

Hence, a decision was made to initiate therapy with intravenous immunoglobulin (IVIG) at a dose of 0.4 g/kg/day for five consecutive days. However, during the administration of the first dose, the patient developed an anaphylactic reaction, necessitating the immediate discontinuation of IVIG. Subsequently, therapeutic plasma exchange was undertaken, and the patient underwent five sessions over a period of ten days. Given the anticipated need for prolonged ventilatory support, tracheostomy was performed on the fourth day of hospitalization. Supportive management, including deep venous thrombosis prophylaxis, physiotherapy, and meticulous nursing care, was instituted simultaneously.

**Figure 1:**
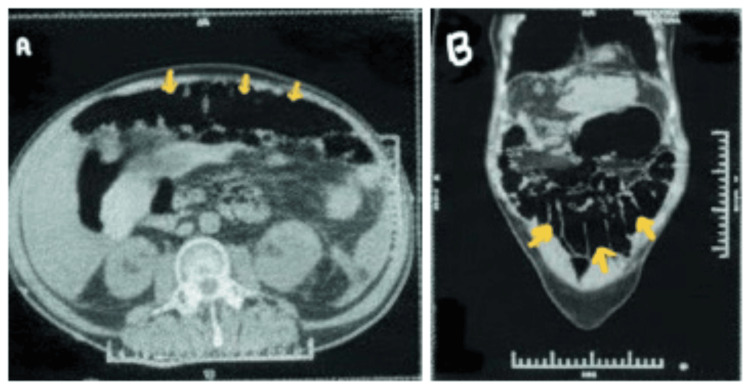
NCCT Abdomen axial and coronal images (A and B) showing dilated large-bowel loops (yellow arrows) without evidence of a transition point, suggestive of acute colonic pseudo-obstruction. NCCT: Non-contrast computed tomography

During the first two weeks of hospitalization, the patient exhibited marked lability of both heart rate and blood pressure, findings consistent with autonomic dysfunction, requiring close hemodynamic monitoring in the intensive care unit. Within ten days of initiating plasmapheresis, she demonstrated improvement in lower limb motor power (MRC grade 2/5) and was able to generate adequate spontaneous tidal volumes. This facilitated successful weaning from mechanical ventilation and transition to oxygen supplementation via a T-piece. However, motor strength in the upper limbs remained severely impaired (MRC grade 1/5). Over time, gradual recovery of motor strength was documented in all four extremities; however, bulbar palsy persisted. After a month of admission, she suddenly developed acute-onset abdominal pain with progressive distension. Non-contrast computed tomography (NCCT) of the abdomen revealed diffuse large-bowel dilatation with air-fluid levels, extending to the rectum without a transition point or wall thickening, findings suggestive of ACPO (Figure 1).

**Figure 2:**
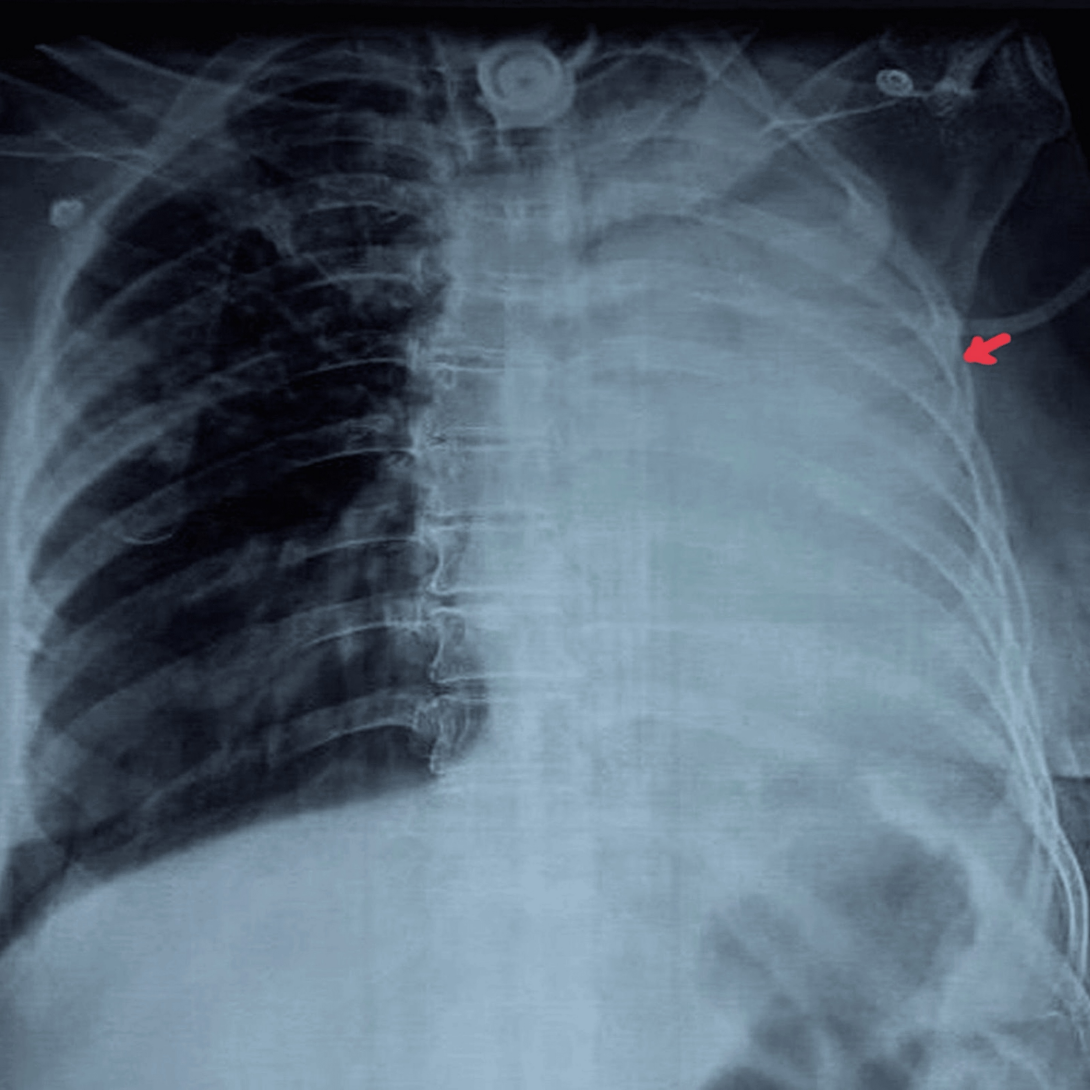
Chest X-ray showing collapse of the left lung (red arrow).

The patient was managed conservatively with prokinetic agents, gastric decompression, and parenteral nutrition, which resulted in resolution of the gastrointestinal manifestations within 3-4 days. However, the persistent bulbar dysfunction delayed decannulation and contributed to an unusually extended duration of intensive care. On the 40th day of hospitalization, she developed sudden desaturation, prompting urgent evaluation. Chest radiography revealed complete collapse of the left lung (Figure 2).

Bedside bronchoscopy was performed, allowing successful removal of obstructing mucus plugs and prompt re-expansion of the lung with rapid improvement in oxygen saturation. Remarkably, it was not until the 80th day of hospitalization that the patient began to show meaningful improvement in bulbar function, with recovery of swallowing capacity sufficient to allow ingestion of liquids without significant difficulty. In light of this clinical recovery, decannulation was successfully performed. Neurological examination at that stage demonstrated motor strength of 4/5 in the lower limbs and 3/5 in the upper limbs, as graded on the MRC scale.

The patient was then finally discharged from the hospital with a comprehensive, individualized rehabilitation program tailored to her neurological deficits. At six months of follow-up, she demonstrated complete neurological recovery, with full restoration of motor strength in all four extremities (MRC grade 5/5). A consolidated overview of the sequential events, diagnostic evaluations, and management strategies is provided in Table 2.

**Table 2:** Timeline of clinical events, diagnostic findings, and interventions during hospitalization. CSF: Cerebrospinal fluid; NCV: Nerve conduction velocity; ACPO: Acute colonic pseudo-obstruction

Hospital Day/Time	Clinical Events	Relevant Diagnostic Study	Intervention
On admission	Dysphagia, bulbar palsy, Upper limb weakness, tachycardia, and hypertension	Routine labs- Normal	Transfer to ICU
8-10 hours	Respiratory distress, quadriplegia	-	Intubation, mechanical ventilation
10-24 hours	Weakness progression	MRI- Normal, CSF- Albumino-cytological dissociation, NCV- suggestive of GBS	Initiation of plasma exchange
Day 4	Anticipation of prolonged ventilation	-	Tracheostomy done
Day 10	Lower limb power improvement, Adequate spontaneous efforts	-	Weaning to T-piece
Day 10-30	Slow improvement in motor power, bulbar palsy persistent	-	Tracheostomy tube kept in situ, Routine ICU care, including physiotherapy.
Day 30	Abdominal pain and distension	NCCT Abdomen- suggestive of ACPO	Conservative management with prokinetics and gastrointestinal decompression
Day 40	Sudden oxygen desaturation	CXR- Left lung collapse	Bedside bronchoscopy
Day 80	Improvement in swallowing capacity and recovery of bulbar palsy	-	Decannulation of tracheostomy tube
Day 84		-	Discharge from hospital
6-month follow-up	Complete recovery of muscle power	-	Rehabilitation complete

## Discussion

This case illustrates an atypical presentation of GBS, underscoring the diagnostic challenges encountered when the course deviates from the classic description. Recognizing such variants requires a high index of suspicion, and it is particularly important because descending forms frequently manifest with early bulbar and respiratory involvement [3,4,7]. These presentations also typically indicate a more severe form of disease and are frequently associated with a prolonged and complex recovery [6]. A structured, stepwise approach that combined clinical evaluation with supportive investigations was pivotal in establishing the diagnosis in our case. Adopting such a systematic strategy is particularly important for atypical variants of GBS, as it facilitates early recognition and timely initiation of therapy [1,6]. The history of antecedent diarrheal illness two weeks prior to symptom onset provided a classic immune-mediated trigger [2]. Neuroimaging with MRI excluded any central nervous system pathology, while CSF analysis demonstrated elevated protein with normal cell count, a hallmark supportive feature of GBS [1,6]. Electrophysiological studies further revealed demyelinating changes, consolidating the diagnosis [8]. Collectively, these multimodal findings supported the identification of an atypical descending variant of GBS and allowed confident differentiation from alternative diagnoses such as myasthenia gravis, botulism, or brainstem pathology [4].

A feature that reinforced the atypical nature of this case was the presence of bulbar dysfunction at onset. Although bulbar involvement develops in up to half of patients with GBS, it more commonly appears later in the disease course, typically after the progression of limb weakness [9]. Its appearance at presentation is therefore distinctly uncommon. In our patient, bulbar weakness preceded or coincided with limb weakness, leading to impaired gag and cough reflexes and necessitating airway protection within the first day of hospitalization. The requirement for ventilatory support at such an early stage underscores the severity of disease in descending or bulbar-predominant variants and is consistent with reports describing their higher likelihood of respiratory failure [3,10].

Beyond bulbar dysfunction, another striking feature in our patient was the extent of autonomic involvement. The autonomic involvement extended beyond cardiovascular instability to the gastrointestinal system, where she developed ACPO (Ogilvie’s syndrome). While gastrointestinal dysmotility and paralytic ileus are occasionally reported in severe GBS, true pseudo-obstruction remains a rare manifestation [11,12].

Most published cases describe its onset within the first one to two weeks after the onset of neurological symptoms, typically during the acute phase when weakness is maximal and ventilatory support is required [13]. In contrast, our patient developed colonic pseudo-obstruction much later - nearly one month after disease onset - making the presentation particularly unusual. This delayed occurrence underscores that gastrointestinal autonomic complications may emerge not only in the acute phase but also during later stages of the illness, thereby highlighting the need for continued vigilance throughout the course of hospitalization [11,13]. Prompt radiologic evaluation and conservative management in this case led to resolution, preventing life-threatening complications such as ischemia or perforation [12].

Collectively, the early and persistent bulbar involvement, pronounced cardiovascular instability, and delayed onset of ACPO highlight the overall severity and clinical complexity of this patient’s disease trajectory. Despite early initiation of immunotherapy (IVIG attempted, then plasma exchange) and comprehensive supportive care, neurological recovery was exceptionally slow. Persistent bulbar dysfunction delayed decannulation until day 80, prolonging ICU stay. Whereas most typical GBS cases treated early demonstrate substantial recovery within weeks to a few months, this patient’s recovery extended over nearly six months [14]. Such protracted trajectories are more frequently observed in patients with poor prognostic markers, including advanced age, need for mechanical ventilation, autonomic dysfunction, and complications such as colonic pseudo-obstruction [8,10,12].

This case, therefore, underscores several important clinical lessons. First, atypical presentations such as descending or bulbar-predominant variants must be recognized early, as they often indicate more severe disease [3,4,7]. Hence, a structured diagnostic approach that combines meticulous clinical evaluation with supportive investigations is vital for early recognition and prompt management of atypical variants [1,6]. Second, autonomic dysfunction in GBS can be multisystemic, extending beyond cardiovascular instability to include rare but serious gastrointestinal complications [5,11]. Third, even with timely therapy, severe variants may follow a prolonged course, necessitating sustained multidisciplinary care and rehabilitation [6,14].

## Conclusions

Our case is unique in that the patient’s presentation did not follow the classic ascending pattern of GBS, instead manifesting as a descending variant with early bulbar involvement, autonomic instability, and delayed ACPO. Although original NCV tracings and antiganglioside antibody testing were unavailable, the preceding diarrheal illness, along with supportive electrophysiological and CSF findings, provided strong diagnostic confidence. Because atypical variants can delay recognition and treatment, maintaining a high index of suspicion and employing a systematic diagnostic approach are essential. Early diagnosis and timely initiation of therapy remain the cornerstones of preventing complications and improving outcomes in such severe presentations.
